# Validation of the indication for colposcopy proposed by the 2019 ASCCP risk-based management consensus guidelines: A single-center study in China

**DOI:** 10.1371/journal.pone.0253493

**Published:** 2021-07-01

**Authors:** Ting Gui, Zhiyan Chen, Fei Chen

**Affiliations:** Department of Obstetrics and Gynecology, National Clinical Research Center for Obstetrics & Gynecologic Diseases, Peking Union Medical College Hospital, Peking Union Medical College, Chinese Academy of Medical Sciences, Beijing, China; National Cancer Institute, UNITED STATES

## Abstract

**Objective:**

To validate the colposcopy indication proposed by the 2019 ASCCP Risk-Based Management Consensus Guidelines for abnormal cervical cancer screening tests (the 2019 ASCCP guidelines).

**Methods:**

Clinical data of 1404 patients who underwent colposcopy in single center in China were reviewed. Based on history and current cervical screening (HPV & cytology), corresponding recommendations were given according to the 2019 ASCCP guidelines. The agreement and discrepancy of colposcopy indication were analyzed between the Chinese consensus and the 2019 ASCCP guidelines.

**Results:**

Colposcopy indication was matched in about 80% patients. The left 20% were recommended with follow-up by the 2019 ASCCP guidelines. The discrepancy mainly focused on patients having a current result of HPV-positive NILM without unknown history. The ratio of observed CIN3+ in our database over estimated CIN3+ by the 2019 ASCCP guidelines was 6.2 (31/5). The ratio was even higher in patients with HPV16/18-positive NILM (7, 28/4), compared with those with other types of high-risk HPV-positive NILM (3, 3/1). The 2019 ASCCP guidelines had a relatively high sensitivity (83.1%), a low specificity (21.5%), a low positive predictive value (14.1%) and a high negative predictive value (89.1%) for prediction of CIN 3+.

**Conclusions:**

We could try to apply the 2019 ASCCP guidelines in Chinese population. The classification of HR-HPV was strongly recommended during risk assessment. For patients with HPV16/18 infection, colposcopy should be recommended. Perspective multi-center randomized controlled trial with reliable follow-up should be performed in the future to confirm the feasibility.

## Introduction

Cervical cancer is the most common malignancy among women in China. The incidence rate of cervical cancer is decreasing in recent years largely due to effective screening. When abnormalities are identified during screening, colposcopy will be adopted to further confirm any cervical lesions. Colposcopy plays a role of connecting bridge between screening and final treatment. The number of unnecessary blinded biopsies and conization has been reduced substantially due to the application of colposcopy. However, colposcopy examination seems to be overused in recent years in China. Appropriate application of colposcopy has great clinical and economic significance. From a public health perspective, reduction in the number of unnecessary invasive procedures will definitely be beneficial for both patients and the society as a whole.

The screening guidelines for cervical cancer continue to be reevaluated and updated, with the overall goal of improving diagnosis and economizing time and resources. The original consensus guidelines was proposed by American Society of Colposcopy and Cervical Pathology (ASCCP) in 2001 [1], and was subsequently updated in 2006 [2] and 2012 [3]. The 2019 ASCCP guidelines are the fourth version for management of cervical cancer screening abnormalities [4]. The 2012 consensus guidelines introduced the principle of “equal management for equal risk”, which was a conceptual breakthrough. Specifically, patients with similar risks for pre-cancer lesions or cancer could be managed similarly, in despite of the variety of screening time points and methods [5]. With a more in-depth understanding of how previous results affect the risk, assessement, the key difference between 2019 ASCCP guidelines and previous versions was the change from “test results-based algorithms” to “algorithms based on quantitative risk estimate values”, officially defined as clinical action thresholds (CATs) [4]. For instance, colposcopy is recommended for patients with HPV-positive and cytology result ≥ atypical squamous cells of undefined significance [ASC-US] by previous guidelines. According to the 2019 ASCCP guidelines, colposcopy should be recommended to patients whose screening results (considering both previous and current) yielded a > 4.0% probability of identifying cervical intraepithelial neoplasm 3+ (CIN3+). Risk estimates used in 2019 ASCCP guidelines were generated from a prospective longitudinal cohort of over 1.5 million patients with over ten-year follow-up at Kaiser Permanente Northern California (KPNC) [6]. The feasibility of these risk estimates in other regions in United States has been validated in other data sets from several screening programs and clinical trials [7]. However, there is no relative data in Asian countries including China.

Therefore, the aims of the present study were to 1) compare the indication for colposcopy in Chinese consensus and the clinical action threshold for colposcopy adopted by the 2019 ASCCP guidelines; 2) assess the applicability of the clinical action threshold for colposcopy adopted by the 2019 ASCCP guidelines in China.

## Materials and methods

### Study design

We retrospectively reviewed the medical records of women who underwent colposcopic examination and cervical biopsy at the colposcopy clinic of Peking Union Medical College Hospital (PUMCH) in China between June 2018 and Jan 2020, and then further assessed the applicability of the clinical action threshold for colposcopy adopted by the 2019 ASCCP guidelines in our Chinese database.

### Subjects

Women included in our research had no history of pelvic radiation or hysterectomy, no sexual intercourse for 3 days before the examination, and no confirmed or clinically suspected immunosuppression or other chronic disease that might have compromised their immune system.

Patients’ information possibly related to cervical intraepithelial lesions were collected, including age, gravidity, parity, number of sex partner, age of first sex intercourse, menstrual status, method of contraception, clinical manifestations including vaginal bleeding and increased vaginal discharge, cervical surgery history, types of transformation zone and the screening results of HPV and cytology.

All data in this study were collected from the hospital’s archived database. This study did not influence the diagnosis or treatment of the patients. This study was approved by the ethics committee of PUMCH. Since all data were deidentified, written informed consent was not necessary due to the retrospective nature of the study.

### Colposcopy indication after abnormal screening in Chinese consensus

1. Continuous high-risk HPV infection + NILM (annual visit >2 consecutive years)
2. HPV-16/18 infection + NILM
3. HPV-positive +ASC-US
4. Abnormal cytological test results greater than ASC-US including LSIL, ASC-H, HSIL, ASC, AGC, invasive cervical cancer

### Clinical action threshold leading to recommendation of colposcopy in 2019 ASCCP risk-based management consensus guidelines [4]

*Guidelines*: Colposcopy is recommended to patients having an immediate risk of CIN3+ of 4.0% or greater, considering both history and current results.

*Rationale*: Among patients referred directly to colposcopy, the immediate risk of CIN 3+ was reported to range from 3%-7% [8–11]. The clinical action threshold for colposcopy referral, a 4% immediate risk of CIN3+, was proposed after balancing the benefits and harms. For patients without screening history, only those with HPV-positive and ASC-US or LSIL cytology would be referred to colposcopy, while patients with HPV-positive and NILM cytology would not.

### Colposcopy and biopsy procedure

All colposcopies were performed by two expert colposcopists and were recorded by using the VIZ-YD system. This was a video exoscope-based system (optical electronic integration colposcopy, Beijing SWSY technology Co., Ltd, China), allowing full high density video documentation of the colposcopic examination process.

Steps in colposcopic assessment of the cervix included the following: 1) clean the cervix with normal saline; 2) assess the cervix about 1 min after the application of 5% acetic acid, and observe margins of the lesion, epithelial color and vascular patterns; and 3) assess the cervix after the application of diluted Lugol’s iodine solution, and observe iodine staining.

All colposcopically detected abnormal areas were biopsied. If the colposcopic examination found no cervix lesions, biopsy specimens would be obtained randomly at the squamo-columnar junction in four quadrants at 3, 6, 9, or 12 o’clock. An endocervical curettage was performed after the cervical biopsy. All biopsy specimens were fixed in formaldehyde and embedded in paraffin routinely.

### Statistical analyses

Continuous variables were recorded as mean±standard deviation (SD) if normally distributed, or median with interquartile range if not normally distributed. Categorical variables were expressed in terms of frequency and percentage. Continuous variables were compared by using the student’s *t* test. Frequencies were compared using the χ2 test or Fisher’s exact test. Univariate analysis was used to analyze the effect of each variable on susceptibility of CIN3+, and variables with p < 0.05 in univariate analysis were adopted for further multivariate analysis. The diagnostic performances were analyzed and compared in terms of sensitivity, specificity, positive predictive value (PPV) and negative predictive value (NPV). The receiver operating characteristic (ROC) curves and the areas under the ROC curves (AUC) were calculated by the 2×2 contingency table and chi-square test, and McNemar test was used to compare the differences in performance. All data were analyzed by using SPSS 23.0 version software (IBM, USA), and p<0.05 was considered statistically significant.

## Results

### Flowchart of patients (Fig 1)

From Jun 2018 to Dec 2019, a total of 1927 patients underwent colposcopy in PUMCH in China. During the process of data preparation, we found 159 patients did not have complete HPV & cytology data record (current and history), 294 patients missed pathological results, 16 patients were diagnosed with cervical cancer and underwent radical surgery or radiation, and 54 patients received hysterectomy for other diseases. Finally, 1404 patients were included in our research, among whom 1215 patients were diagnosed as inflammation or CIN 1 or CIN 2, and 189 patients were diagnosed as CIN3+ (CIN 3 or adenocarcinoma in situ of cervicx (AIS) or invasive cervical cancer).

**Fig 1:**
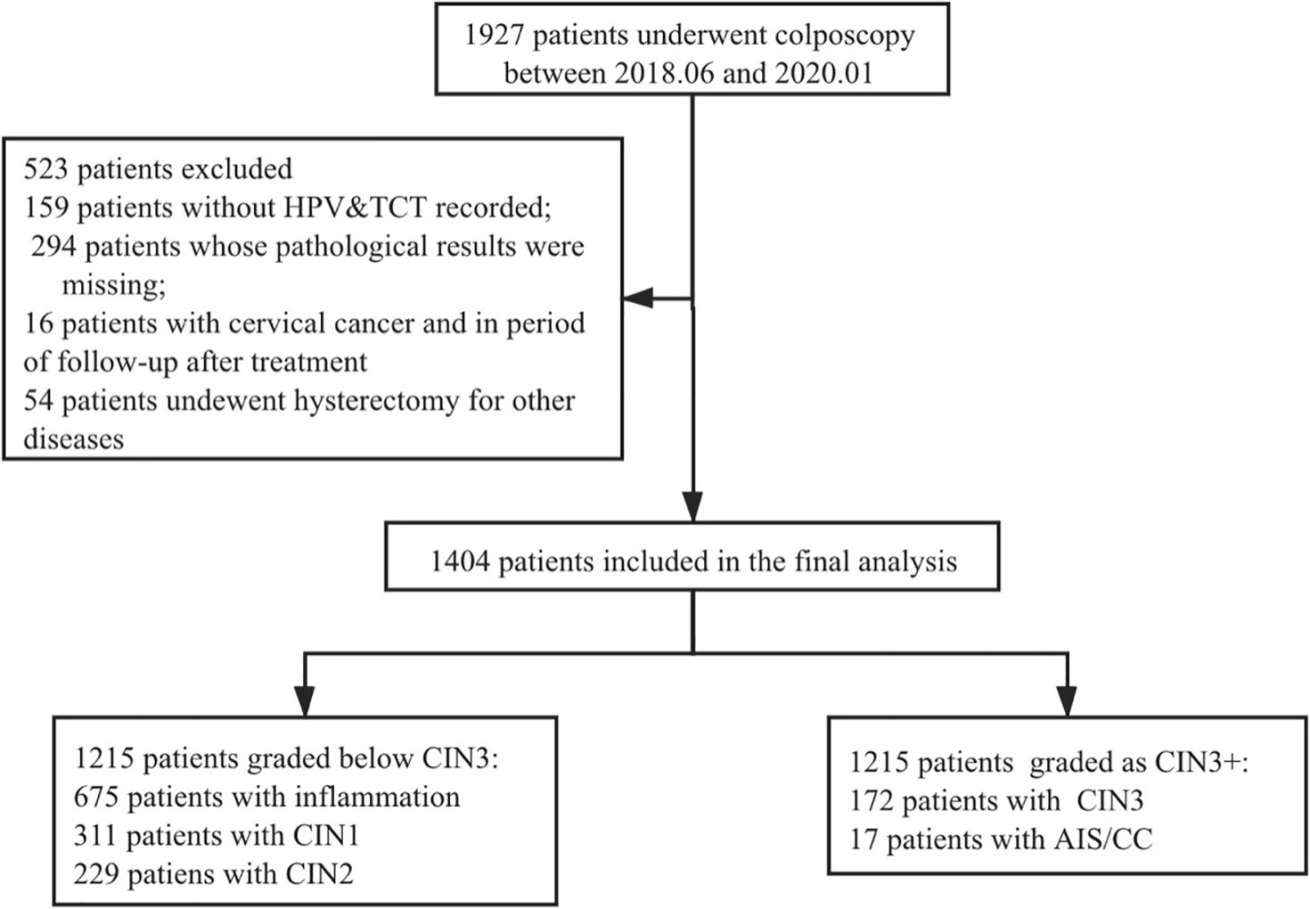
Flowchart of patients. Abbreviations: HPV, human papillomavirus; TCT, liquid-based cytology test; CIN, cervical intraepithelial neoplasia; CIN3+, CIN3/AIS/CC; AIS, adenocarcinoma in situ; CC, invasive cervical cancer.

### Basic characteristics of patients (Table 1)

Patients included in our research were classified into two groups according to the final pathological diagnosis after colposcopy examination and biopsy, the < CIN3 group and the ≥ CIN3+ group.

**Table 1:** Basic characteristics of patients.

Variable	<CIN3+	≥CIN3	p-value
N	1215	189	
Age	40.6 ± 10.5	39.5 ± 11.1	NS
Gravidity	2.0 ± 1.8	2.2 ± 1.7	NS
Parity	0.9 ± 0.8	1.0 ± 0.8	NS
No. of sex partners	1.8 ± 1.8	2.2 ± 2.6	0.046
Age of first sex	22.5 ± 3.2	21.5 ± 3.6	0.014
Menstrual status			NS
Premenopausal period	963 (80.6%)	148 (81.3%)	
Lactation period	4 (0.3%)	2 (1.1%)	
Menopause	228 (19.1%)	32 (17.6%)	
Contraception method			NS
No	404 (36.6%)	58 (33.7%)	
COC	6 (0.5%)	2 (1.2%)	
Condom	624 (56.5%)	102 (59.3%)	
IUD	71 (6.4%)	10 (5.8%)	
Vaginal bleeding	119 (10.0%)	19 (10.4%)	NS
Increased vaginal discharge	6 (0.5%)	1 (0.5%)	NS
Cervical surgical history	153 (12.6%)	17 (9.0%)	NS
The transformation Zone			NS
TZ-1	471 (39.4%)	68 (37.4%)	
TZ-2	386 (32.3%)	65 (35.7%)	
TZ-3	337 (28.2%)	49 (26.9%)	
Previous HPV			<0.001
HPV-	19 (1.8%)	3 (1.8%)	
Unknown	610 (58.3%)	117 (68.8%)	
HPV others+	292 (27.9%)	18 (10.6%)	
HPV 16/18+	126 (12.0%)	32 (18.8%)	
Previous TCT			0.050
NILM	219 (25.9%)	22 (15.8%)	
Unknown	604 (71.4%)	115 (82.7%)	
ASC-US	11 (1.3%)	1 (0.7%)	
LSIL	12 (1.4%)	1 (0.7%)	
Present HPV			<0.001
HPV-	53 (4.4%)	5 (2.6%)	
HPV others+	667 (55.0%)	57 (30.2%)	
HPV 16/18+	493 (40.6%)	127 (67.2%)	
Present TCT			<0.001
NILM	450 (37.2%)	48 (25.4%)	
ASC-US	338 (27.9%)	29 (15.3%)	
LSIL	345 (28.5%)	35 (18.5%)	
ASC-H	24 (2.0%)	20 (10.6%)	
AGC	18 (1.5%)	1 (0.5%)	
HSIL	36 (3.0%)	56 (29.6%)	

**Abbreviations**: COC = combined contraceptive pills. IUD = intrauterine device. NS = no statistically significant. TZ = Transformation zone. NILM = negative for intraepithelial lesion or malignancy. ASC-US = atypical squamous cells of undetermined significance. LSIL = low-grade squamous intraepithelial lesion. ASC-H = atypical squamous cells, cannot exclude high-grade squamous intraepithelial lesion. AGC = Atypical glandular cells. HSIL = high-grade squamous intraepithelial lesion. AIS = adenocarcinoma in situ.

**Note:** The column of “<CIN3+” indicates patients whose pathological results were graded lower than CIN3, including inflammation, CIN1 and CIN2. The column of “≥CIN3+” represents patients whose pathological results were graded CIN 3 or greater, including CIN3, AIS and invasive cervical cancer.

Our research showed that CIN3+ rates were higher in patients who had more sex partners and had their first sexual intercourse in younger age, with p = 0.046 and p = 0.014, respectively. CIN3+ rates were also higher in patients with HPV infection, especially HPV16/18 infection (p < 0.001, for both previous and current infection), and with higher grade of cytology result (p = 0.05 and p < 0.001 for previous and current cytology status, respectively).

However, statistical analyses showed no significant differences between the two groups in the following terms including the patients’ age, the gravidity, the parity, the menstruation status, the contraception method, and the cervical surgery history. In addition, no significant differences were observed regarding the clinical manifestations and the status of transformation zone of cervix.

### Univariate analyses of high-risk factors for CIN 3+ (Table 2)

Univariate analyses showed that patients with present HPV 16/18 infection had a nearly threefold increased risk of CIN3+ compared with the patients with a negative HPV status (OR = 2.73, 95%CI [1.07–6.97]). Furthermore, patients with present cytology of ASC-H and HSIL increased the CIN3+ risk by nearly eight-fold (OR = 7.81, 95%CI [4.02–15.17]) and fifteen-fold (OR = 14.58, 95%CI [8.72–24.38]), compared with patients who had normal cytology screening test.

**Table 2:** Univariate analysis of factor associated with CIN3+.

	Odd ratio	95% CI	p-value
Age	0.99	0.98, 1.00	NS
Gravidity	1.06	0.98, 1.14	NS
Parity	1.18	0.98, 1.43	NS
No. of sex partner	1.08	0.99, 1.17	NS
Age of first sex	0.91	0.86, 0.97	0.004
Menstrual status			
Premenopausal period	1	-	-
Lactation period	3.25	0.59, 17.92	NS
Menopause	0.91	0.61, 1.37	NS
Contraception method			
No	1	-	-
COC	2.32	0.46, 11.78	NS
Condom	1.14	0.81, 1.61	NS
IUD	0.98	0.48, 2.01	NS
Vaginal bleeding	1.05	0.63, 1.75	NS
Increased vaginal discharge	1.07	0.13, 8.95	NS
Cervical surgical history	0.69	0.41, 1.16	NS
Previous HPV			
HPV-	1	-	-
Unknown	1.21	0.35, 4.17	NS
HPV+	0.76	0.22, 2.65	NS
HPV others+	0.43	0.09, 2.05	NS
HPV 16/18+	1.78	0.38, 8.22	NS
Previous TCT			
NILM	1		
Unknown	1.90	1.17, 3.07	0.009
ASC-US	0.90	0.11, 7.34	NS
LSIL	0.83	0.10, 6.68	NS
Present HPV			
HPV-	1	-	-
HPV+	1.68	0.66, 4.25	NS
HPV others+	0.91	0.35, 2.36	NS
HPV 16/18+	2.73	1.07, 6.97	0.036
Present TCT			
NILM	1	-	-
ASC-US	0.80	0.50, 1.30	NS
LSIL	0.95	0.60, 1.50	NS
ASC-H	7.81	4.02, 15.17	<0.001
AGC	0.52	0.07, 3.99	NS
HSIL	14.58	8.72, 24.38	<0.001

**Abbreviations**: COC = combined contraceptive pills. IUD = intrauterine device. NS = no statistically significant. TZ = Transformation zone. NILM = negative for intraepithelial lesion or malignancy. ASC-US = atypical squamous cells of undetermined significance. LSIL = low-grade squamous intraepithelial lesion. ASC-H = atypical squamous cells, cannot exclude high-grade squamous intraepithelial lesion. AGC = Atypical glandular cells. HSIL = high-grade squamous intraepithelial lesion. AIS = adenocarcinoma in situ. 95%CI = 95% confidence interval. AIS = adenocarcinoma in situ. CIN3+ = CIN3 /AIS/cervical cancer.

**Note:** The column of “<CIN3+” indicates patients whose pathological results were graded lower than CIN3, including inflammation, CIN1 and CIN2. The column of “≥CIN3+” represents patients whose pathological results were graded CIN 3 or greater, including CIN3, AIS and invasive cervical cancer.

### Multivariate analyses of high-risk factors for CIN 3+ (Table 3)

All variates with P < 0.05 in the univariate analyses were included in the multivariate regression analysis. Patients, who were infected with other types of high-risk HPV(HR-HPV) and HPV 16/18, showed an increased risk for CIN3+ (OR = 5.81,0R = 16.93, respectively), although both of which were not statistically significant. After adjusting for age, gravidity, parity, number of sex partner, menstruation status and contraception method, patients with current cytology of ASC-H and HSIL had a twenty-fourfold (adjusted OR = 23.90, 95%CI [5.50–103.92]) and approximate ten-fold (adjusted OR = 10.32, 95%CI [4.10–25.99]) risk for CIN3+, compared with patients who had a normal cytology test.

**Table 3:** Multivariate analysis of factor associated with CIN3+.

Variable	Odd ratio	95% CI	P-value
Age of first sex	0.91	0.84, 0.99	0.0258
Previous HPV			
HPV-	1.0	-	-
Unknown	inf.	0.00, Inf	NS
HPV others+	1005500.74	0.00, Inf	NS
HPV 16/18+	1651122.49	0.00, Inf	NS
Previous TCT			
NILM	1.0	-	-
Unknown	0.00	0.00, Inf	NS
ASC-US	0.00	0.00, Inf	NS
LSIL	0.00	-	NS
Present HPV			
HPV-	1.0	-	-
HPV others+	5.81	0.31, 110.63	NS
HPV 16/18+	16.93	0.86, 333.25	NS
Present TCT			
NILM	1.0	-	-
ASC-US	1.08	0.52, 2.25	NS
LSIL	1.34	0.61, 2.95	NS
ASC-H	26.39	7.29, 95.44	<0.0001
AGC	19.01	0.83, 434.11	NS
HSIL	9.87	4.24, 22.95	<0.0001

**Abbreviation**: Inf = infinite. NILM = negative for intraepithelial lesion or malignancy. ASC-US = atypical squamous cells of undetermined significance. LSIL = low-grade squamous intraepithelial lesion. ASC-H = atypical squamous cells, cannot exclude high-grade squamous intraepithelial lesion. AGC = Atypical glandular cells. HSIL = high-grade squamous intraepithelial lesion. AIS = adenocarcinoma in situ. 95%CI = 95% confidence interval.

### Risk estimates and management recommendation according to the 2019 guidelines (Tables 4 and 5)

In our study, a total of 1404 patients received colposcopy examination due to cervical screening abnormality in PUMCH in accordance with the Chinese consensus. According to the risk estimate tables supporting the 2019 ASCCP guidelines [6] and the management recommendation flow [4, Fig 1], our research found that the immediate CIN3+ risk and the 5-year CIN3+ risk were both significantly higher in the ≥ CIN3+ group compared with the < CIN 3 group (p<0.001 both). Specifically, 1 of 1404 (0.07%) patient was recommended 5-y regular screening, 1 of 1404 (0.07%) patient was recommended 3-y regular screening, 291 of 1404 (20.66%) patients were recommended 1-y regular screening, and 1111 of 1404 (79.20%) patients were recommended colposcopy/immediate treatment. Among the 293 patients recommended for follow-up, 261 (89.07%) patients were graded lower than CIN3 (166 inflammation, 47 CIN 1, 48 CIN 2), while 32 (10.93%) patients were graded CIN3+ (26 CIN3, 6 AIS/cancer).

**Table 4:** Risk estimates and recommended managements.

Variable	<CIN3+^a^	≥CIN3^b^	P-value
N	1215	189	
Immediate CIN3+ risk			<0.001
5-year CIN3+ risk	4.3 (4.1–5.0)	5.4 (4.1–44.0)	<0.001
Recommended management	7.2 (6.9–8.5)	9.5 (6.9–50.0)	<0.001
5-y follow up	1 (0.1%)	0 (0.0%)	
3-y follow up	1 (0.1%)	0 (0.0%)	
1-y follow up	259 (21.3%)	32 (16.9%)	
Colposcopy	905 (74.5%)	85 (44.9%)	
Treatment/colposcopy	48 (4.0%)	72 (38.1%)	
Treatment preferred	1 (0.1%)	0 (0.0%)	
Pathology			<0.001
Inflammation	675 (55.6%)	0 (0.0%)	
CIN1	311 (25.6%)	0 (0.0%)	
CIN2	229 (18.8%)	0(0.0%)	
CIN3	0(0.0%)	172 (91.0%)	
AIS/Cancer	0 (0.0%)	17 (9.0%)	

**Abbreviations**: CIN = cervical intraepithelial neoplasia. AIS = adenocarcinoma in situ.

CIN3+ = CIN3 /AIS/cervical cancer.

**Note:** The column of “<CIN3+” indicates patients whose pathological results were graded lower than CIN3, including inflammation, CIN1 and CIN2. The column of “≥CIN3+” represents patients whose pathological results were graded CIN 3 or greater, including CIN3, AIS and invasive cervical cancer.

**Table 5:** Agreement between recommended management according to the 2019 ASCCP guidelines and final cervical histopathologic diagnoses (%).

Recommended management		Cervical histopathologic diagnoses					Total
		Inflammation	CIN1	CIN2	CIN3	AIS/CC	
	5-y follow up	1(100)	0(0)	0(0)	0(0)	0(0)	1(100)
	3-y follow up	1(100)	0(0)	0(0)	0(0)	0(0)	1(100)
	1-y follow up	164(56.3)	47(16.2)	48(16.5)	26(8.9)	6(2.1)	291(100)
	Colposcopy	498(50.3)	253(25.6)	154(15.6)	81(8.2)	4(0.4)	990(100)
	Treatment/colposcopy Treatment preferred	11(9.2) 0(0)	11(9.2) 0(0)	26(21.7) 1(100)	65(54.2) 0(0)	7(5.8) 0(0)	120(100) 1(100)
**Tota**l		675	311	229	172	17	1404

**Abbreviations**: CIN = cervical intraepithelial neoplasia. AIS = adenocarcinoma in situ. CIN = cervical intraepithelial neoplasia. CC = cervical cancer.

### Comparison of management recommendation between Chinese consensus and 2019 ASCCP guidelines (Tables 6 and 7)

About 80% of the patients admitted to colposcopy clinic in PUMCH in China were also recommended colposcopy procedure according to the 2019 ASCCP guidelines, while the left 20% were recommended follow-up, among whom 32 patients were graded as CIN 3+ accounting for 2.3% of the total study population.

**Table 6:** Portability of KPNC risks and risk-based management to the PUMCH data.

Table	PUMCH^a^			KPNC^b^		0/E	Recommended management
	n	%	O.CIN3+	Immed.risk	E.CIN3+		
**Table 1A**	**725**	0.52	**117**		**60**		
**HPV-**	**32**	0.02	**3**		**3**		
NILM	1	0.00	0	0.00	0	0/0	5-y follow up
ASC-US	1	0.00	0	0.04	0	0/0	3-y follow up
LSIL	14	0.01	0	1.1	0	0/0	1-y follow up
ASC-H	6	0.00	0	3.4	0	0/0	Colposcopy[s]
HSIL+	10	0.01	3	25	3	1	Treatment/colposcopy
**HPV+**	**693**	0.49	**114**		**57**		
NILM	228	0.16	31	2.1	5	6.2	1-y follow up
ASC-US	196	0.14	16	4.4	9	1.78	Colposcopy
LSIL	185	0.13	17	4.3	8	2.13	Colposcopy
ASC-H	24	0.02	15	26	6	2.5	Treatment/colposcopy
AGC	1	0.00	0	26	0	0/0	Treatment/colposcopy
HSIL	59	0.04	35	49	29	1.21	Treatment/colposcopy
**Table 1B**	**9**	0.01	**0**		**0**		
**HPV-**	**3**	0.00	**0**		**0**		
LSIL	1	0.00	0	0.4	0	0/0	1-y follow up
ASC-H	2	0.00	0	2.8	0	0/0	Colposcopy[s]
**HPV+**	**6**	0.00	**0**		**0**		
NILM	3	0.00	0	0.7	0	0/0	1-y follow up
ASC-US	2	0.00	0	2	0	0/0	1-y follow up
LSIL	1	0.00	0	2.1	0	0/0	1-y follow up
**Table 2A**	**4**	0.00	**1**		**0**		
**HPV-**	**2**	0.00	**1**		**0**		
LSIL	1	0.00	0	2.4	0	0/0	1-y follow up
HSIL	1	0.00	1	11	0	1/0	Colposcopy
**HPV+**	**2**	0.00	**0**		**0**		
LSIL	2	0.00	0	2.6	0	0/0	1-y follow up
**Table 2B**	**9**	0.01	**2**		**1**		
**HPV-**	**5**	0.00	**1**		**0**		
ASC-US	1	0.00	0	0	0	0/0	1-y follow up
LSIL	3	0.00	1	0	0	1/0	1-y follow up
HSIL	1	0.00	0	0	0	0/0	Colposcopy[s]
**HPV+**	**4**	0.00	**1**		**1**		
LSIL	2	0.00	0	7.9	0	0/0	Colposcopy
HSIL	2	0.00	1	33	1	1	Treatment/colposcopy
**Table 2C**	**461**	0.33	**50**		**31**		
**HPV-**	**9**	0.01	**0**		**0**		
NILM	1	0.00	0	0.74	0	0/0	1-y follow up
LSIL	6	0.00	0	2.3	0	0/0	1-y follow up
AGC	2	0.00	0	8.3	0	0/0	colposcopy
**HPV+**	**452**	0.32	**50**		**31**		
NILM	198	0.14	15	4.1	8	2	Colposcopy
ASC-US	109	0.08	8	5.4	6	1.33	Colposcopy
LSIL	117	0.08	12	5	6	2	Colposcopy
ASC-H	7	0.00	2	22	2	1	Colposcopy
AGC	3	0.00	0	33	1	0/1	Colposcopy
HSIL	18	0.01	13	44	8	1.63	Treatment/colposcopy
**Table 4A**	**26**	0.02	**0**		**0**		
**HPV-**	**2**	0.00	**0**		**0**		
ASC-US/LSIL	1	0.00	0	0.05	0	0/0	1-y follow up
High grade	1	0.00	0	1.6	0	0/0	colposcopy
**HPV+**	**24**	0.02	**0**		**0**		
NILM	11	0.01	0	2.1	0	0/0	1-y follow up
ASC-US/LSIL	13	0.01	0	3.1	0	0/0	1-y follow up
**Table 4B**	**2**	0.00	**0**		**1**		
**HPV+**	**2**	0.00	**0**		**1**	0	
High grade	2	0.00	0	28	1	0/1	colposcopy
**Table 5A**	**168**	0.12	**19**		**20**		
**HPV-**	**5**	0.00	**0**		**0**		
NILM	1	0.00	0	0.03	0	0/0	1-y follow up
ASC-US/LSIL	3	0.00	0	0.75	0	0/0	1-y follow up
High grade	1	0.00	0	18	0	0/0	colposcopy
**HPV+**	**163**	0.12	**19**		**20**		
NILM	59	0.04	2	5.8	3	0.67	colposcopy
ASC-US/LSIL	89	0.06	10	10	9	1.11	colposcopy
High grade	15	0.01	7	53	8	0.88	Treatment/colposcopy
**Total**	**1404**	1.00	**189**		**113**		

**Note:** High grade corresponds to ASC-H, AGC, HSIL+. PUMCH^a^ corresponds to PUMCH data. KPNC^b^ corresponds to risks and risk-based management according to KPNC cohort. The column named “table” represents the risk-based management tables used to assess the risk of CIN3+ from KPNC cohort in the 2019 ASCCP guidelines. “N” indicates number of observations of our data. The third column represents the percentage of observations among all 1404 patients in our study. “Immed.risk” is for immediate CIN3+ risk as estimated from KPNC; The column of “E.CIN3+” represents the expected patients of CIN3+ according to the immediate CIN3+ risk. The column of “O.CIN3+” represents the patients of CIN3+ observed in PUMCH data. 0/E, observed/expected. the last column of “recommended management” represents the different management according to the KPNC-based management consensus.

**Table 7:** Portability of KPNC risks and risk-based management for specific scenario of HPV+ NILM to the PUMCH data.

Table	PUMCH^a^			KPNC^b^		0/E	Recommended management
	n	%	O.CIN3+	Immed.risk	E.CIN3+		
**Table 1A**							
**HPV other+**							
NILM	30	0.02	3	2.1	1	3	1-y follow up
**HPV 16/18+**							
NILM	198	0.14	28	2.1	4	7	1-y follow up
**Total**	228	0.16	31		5		

**Note:** PUMCH^a^ corresponds to PUMCH data. KPNC^b^ corresponds to risks and risk-based management according to KPNC cohort. The column named “table” represents the risk-based management tables used to assess the risk of CIN3+ from KPNC cohort in the 2019 ASCCP guidelines. “N” indicates number of observations of our data. The third column represents the percentage of observations among all 1404 patients in our study. “Immed.risk” is for immediate CIN3+ risk as estimated from KPNC; The column of “E.CIN3+” represents the expected patients of CIN3+ according to the immediate CIN3+ risk. The column of “O.CIN3+” represents the patients of CIN3+ observed in PUMCH data. O/E, observed/expected. the last column of “recommended management” represents the different management according to the KPNC-based management consensus.

The discrepancy mainly focused on one scenario in Table 1A provided by Egemen et al [6], in which patients had a current test result of HPV-positive & NILM and an unknown/undocumented history, with 1-year follow-up recommendation on the basis of immediate CIN3+ risk of 2.1% (< 4.0%) under this circumstance. The ratio of CIN3+ patients observed in PUMCH database over CIN3+ patients expected according to the immediate CIN3+ risk (O/E) in this scenario was 6.2 (31/5). When the HPV genotypes of this subset of patients were further categorized to HPV16/18 and 12 other genotypes of high-risk HPV (other HR-HPV), the CIN3+ ratio was even higher in patients with the result of HPV16/18-positive NILM (O/E = 7, 28/4), compared with patients with the result of other HR-HPV-positive NILM (O/E = 3, 3/1).

### Accuracy of management recommendation by the 2019 ASCCP guidelines in predicting Cervical Intraepithelial Neoplasia (CIN) (Table 8)

The sensitivity, specificity, positive predictive value (PPV), and negative predictive value (NPV) for prediction of CIN3+ were shown in Table 8. The 2019 ASCCP guidelines had a relatively high sensitivity (83.1%, 95% CI [77.0%-88.1%]) and a low specificity (21.5%, 95% CI [19.2%-23.9%]) for prediction of CIN 3+. Besides, our statistical analyses showed a low PPV (14.1%, 95% CI [12.1%-16.3%]) and a high NPV (89.1%, 95% CI [84.9%-92.4%]) for prediction of CIN 3+.

**Table 8:** Accuracy of management recommendation by the 2019 ASCCP guidelines in predicting cervical intraepithelial neoplasia.

	Result for histopathological diagnoses	
	<CIN3+ vs. CIN3+	<CIN2+ vs. CIN2+
Area under ROC curve (AUC)(95% CI)	0.52 (0.50–0.55)	0.50 (0.58–0.63)
Sensitivity(95% CI)	83.1% (77.0%-88.1%)	10.5% (7.7%-13.9%)
Specificity(95% CI)	21.5% (19.2%-23.9%)	90.1% (88.0%-91.9%)
Positive-predictive value(95% CI)	14.1% (12.1%-16.3%)	31.2% (23.6%-39.6%)
Negative-predictive value(95% CI)	89.1 (84.9%-92.4%)	70.2% (67.6%-72.8%)

In addition, we also analyzed the sensitivity, specificity, PPV and NPV of 2019 ASCCP guidelines in differentiating a cervical lesion of CIN2/3 from CIN 1. Table 8 showed that the sensitivity was 10.5% (CI [7.7%-13.9%]), the specificity was 90.1% (CI [88.0%-91.9%]), the PPV was 31.2% (CI [23.6%-39.6%]), and the NPV was 70.2% (CI [67.6%-72.8%]).

## Discussion

The 2019 ASCCP guidelines highlight that detection and treatment of pre-cancer lesions still remains the main aim of cervical cancer prevention, and this version of guidelines comprehensively use and expand upon the principle of “equal management for equal risks”.

The 2019 ASCCP guidelines take both the current and the previous screening tests into consideration, and make recommendations based on immediate CIN3+ risk which is the probability of patients currently having CIN3+, and 5-year CIN3+ risk which is the probability of developing CIN3+ over the ensuing 5 years. It is worth noting that, CIN 3+, instead of CIN 2+, is chosen as main clinical endpoint for risk estimates, because CIN 3+ is more pathologically reproducible [12], and has a more similar HPV-type distribution to that of invasive cervical cancers [13], while CIN 2 has a greater tendency for regression even without any treatment [14–16].

Statisticians have conducted extensive data analyses effort to produce risk estimates for all combinations of screening tests, mainly based on Kaiser Permanente Northern California (KPNC) database, which was the largest and most comprehensive data set in the United States [6]. Three additional databases were also analyzed to ensure that results would be applicable to patients of diverse racial, ethnic, and socioeconomic strata, including the Onclarity HPV trial [17, 18], the New Mexico State HPV Pap Registry [19, 20], and the US Centers for Disease Control and Prevention’s National Breast and Cervical Cancer Early Detection Program [21].

To date, our research is the first study of investigating the applicability of risk-based recommendation for colposcopy in China, by comparing the colposcopy indication between Chinese consensus and 2019 ASCCP guidelines.

In our study, 1404 patients were included in our research. Patients were classified into two groups, the < CIN3 group (1215 (86.5%)) and the ≥ CIN3+ group (189 (13.5%)). Patients who had more sex partners and had their first sexual intercourse in younger age were more susceptible to CIN3+, concordant with previous published studies. Final multivariate analyses showed patients with current cytology of ASC-H and HSIL had approximate twenty-six fold and ten fold risk for CIN3+ with statistical significance, compared with patients who had a normal cytology test. However, the HPV status (previous and present) and the previous cytology result were not independent risk factors for CIN 3+ statistically. This result could probably be accounted by the selection bias of our research population. Patients selected in our research all had underwent colposcopy procedure, with an overwhelming majority of HPV positive and such a tiny part of HPV negative (32 vs. 3 or 127 vs. 5). Besides, it is widespread accepted that HPV test has relatively higher sensitivity and negative predictive value, while cytology test has relatively higher specificity and positive predictive value.

We further analyzed the applicability of the 2019 ASCCP guidelines for predicting cervical lesions in China. Our results showed that the agreement of colposcopy indication was perfectly matched in about 80% of the patients, suggesting that the clinical action threshold for colposcopy adopted in 2019 ASCCP guidelines was applicable in China in general. Nevertheless, more attention should be paid to the left 20% who received colposcopy examination in PUMCH in China while the 2019 ASCCP guidelines recommended follow-up. Among the 20% patients, 32 patients were graded as CIN 3+ whom might be misdiagnosed subsequently, accounting for about 2.3% of the total study population. The discrepancy mainly focused on patients having a current test result of HPV-positive NILM and an unknown history, with 1-year follow-up recommendation on the basis of immediate CIN3+ risk of 2.1% (< 4.0%). Here we introduced a ratio O/E, that was the number of actually observed CIN3+ patients after final pathological diagnosis over the number of expected CIN3+ calculated by the total number of patients × immediate CIN 3+ risk 2.1%. In our study, the ratio O/E was 6.2 (31/5), suggesting that CIN3+ could be probably misdiagnosed if 1-year follow-up recommendation was given according to the 2019 ASCCP guidelines. Additionally, when the HPV types were further categorized to HPV16/18 and others HR-HPV, the O/E ratio was even higher in patients with HPV16/18-positive NILM, compared with patients with others HR-HPV-positive NILM. Therefore, the classification of HR-HPV was strongly recommended during the risk assessment. For patients with current HPV-positive NILM without prior documented HPV status, 1-year follow-up could be recommended for patients with others HR-HPV infection, but colposcopy should be recommended for patients with HPV16/18 infection.

In the present study, we also found that the 2019 ASCCP guidelines had a high sensitivity (83.1%), capable of differentiating a cervical lesion of CIN 3+ from CIN 1/2; however, the specificity was low (21.5%). This would probably lead to over-estimation and thereby increase patients’ cost. In addition, the 2019 ASCCP guidelines had a low PPV of 14.1% and a high NPV of 89.1%, indicating that if the guidelines do not recommend colposcopy, the probability for a patient of developing CIN 3+ would be about 10%.

In cervical cancer screening, once screening abnormalities are identified, colposcopy would probably be recommended to confirmed any cervical lesions. The primary task for colposcopy examination is to improve the accuracy of diagnosing high-grade lesions and prevent misdiagnosis. Generally, an overestimated risk leads to an unnecessary colposcopy procedure and cervical biopsy, whereas an underestimated risk leads to missed diagnosis, possibly high-grade lesions or even carcinomas.

Sensitivity is the percentage of all patients who test positive. During risk assessment, most clinicians would prefer higher sensitivity, since the significant negative consequence of missing high grade cervical lesions are far more pressing, despite the potential for over-treatment. In addition, with a higher NPV, the likelihood that a test-negative person has no disease, screening-negative women are at a lower risk for developing cervical lesions over multiple years. A high sensitivity and NPV for colposcopy referral maximize the detection of cervical lesions and minimized the misdiagnosis.

In our research, the study population was all patients who had already been referred to colposcopy, and the final pathological diagnosis of high grade cervical lesions was used to evaluate the indication for colposcopy. It is worth noting that, the prevalence of disease in a population has a major impact on the specificity, the percentage of all people without certain disease who test negative. In a referral population, the higher prevalence of disease causes a decrease in specificity. This is due to the increased prevalence, which diminishes the denominator in the calculation for the specificity. Besides, in a referral population, as screening abnormalities are quite common and not all abnormalities would cause disease and have a high tendency for spontaneous regression, the PPV, the likelihood that a test-positive person having disease, would be relatively low.

Therefore, the sensitivity and NPV have greater significance than the specificity and PPV in cervical cancer screening.

Our study had several limitations. First, this research was a single-center study. Second, the selection bias was inevitable. Third, this research was a retrospective review without long-term follow-up. In future study, a perspective multi-center randomized controlled trial with reliable follow-up should be performed.

## Conclusions

We could try to apply the 2019 ASCCP guidelines in Chinese population. The classification of HR-HPV was strongly recommended during risk assessment. For patients with current test result of HPV-positive NILM and without prior documented HPV status, 1-year follow-up could be recommended for patients with others HR-HPV infection, but colposcopy should be recommended for patients with HPV16/18 infection. Perspective multi-center randomized controlled trial with reliable follow-up should be performed in the future.
